# How a Simple Diabetic Ketoacidosis Was Actually a Deadly Liver Abscess

**DOI:** 10.7759/cureus.40891

**Published:** 2023-06-24

**Authors:** Teresa Del Rio, Basilides Fermin, Kala Sury

**Affiliations:** 1 Internal Medicine, Wyckoff Heights Medical Center, Brooklyn, USA; 2 Pulmonary Critical Care, Internal Medicine, Wyckoff Heights Medical Center, Brooklyn, USA

**Keywords:** elevated liver associated enzymes, surgery general, infectious and parasitic diseases, hepatology, pyogenic liver abscesses

## Abstract

Pyogenic liver abscesses (PLA) are rare causes of infection in immunocompetent individuals in developed countries. In this report, we discuss a rare presentation and the risk factors associated with developing PLA. Our aim is to raise awareness about PLA developing in patients with uncommon risk factors, enabling early identification and appropriate treatment. The case involves a male patient who presented to the hospital with generalized weakness, was admitted for diabetic ketoacidosis (DKA), and incidentally had elevated liver enzymes that required further investigation. It is important to note that risk factors such as diabetes mellitus, proton pump inhibitors, and colon malignancies are very rare but have been reported in isolated cases as potential risks for developing PLA. Early diagnosis of PLA is crucial due to its high mortality rate, even with intervention.

## Introduction

Pyogenic liver abscesses (PLA) are an increasing cause of morbidity and mortality worldwide. In North America, the annual incidence of this condition has been reported as 2.3 cases per 100,000 people; this incidence tends to increase with advancing age [1]. Diagnosing PLA is challenging as patients present with non-specific symptoms of fever, right upper abdominal pain, nausea, and vomiting [2-5]. The most common etiologies are attributed to bacterial, parasitic, or mixed fungal infections. These organisms invade and multiply within a healthy, or diseased, liver parenchyma and cause a suppurated cavity known as PLA [1, 4-6]. In patients 60 years or older, the most common cause of PLA has been described as spread through the portal venous system [2]. Co-morbid conditions associated with significantly higher risk for developing PLA include a history of liver transplant, history of malignancy, diabetes mellitus, and proton pump inhibitor (PPI) use [1, 3-4]. In patients with no previous transplant history, colonoscopy is recommended as colonic malignancies invade the mucosa and therefore form a pathway to the portal system. This pathway can lead to a hematogenous spread of common pathogens such as *Klebsiella pneumonia*, which can lead to the formation and spread of septic emboli [6,7]. 

## Case presentation

**Figure 1:**
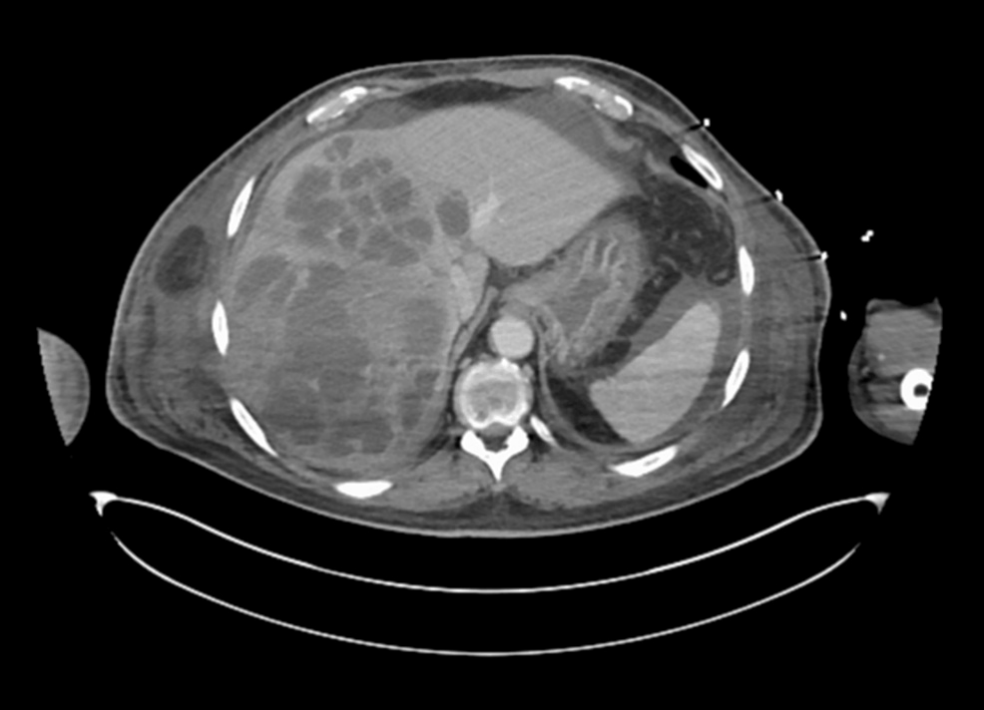
Initial CT scan of the abdomen and pelvis with IV contrast showing multiloculated cystic mass involving the majority of the right lobe of the liver. CT, computed tomography; IV, intravenous.

A 71-year-old man with a medical history of Type 2 diabetes mellitus using insulin, benign prostatic hyperplasia, and hypertension initially presented to the hospital with complaints of generalized body weakness. Physical examination revealed physical wasting and fatigue, but no signs of abdominal tenderness, jaundice, or icteric sclera were observed. On presentation to the emergency room, his blood glucose levels were found to be above 400 mg/dL, and he had an anion gap of 20 mEq/L. He was diagnosed with diabetic ketoacidosis (DKA) and was subsequently admitted to the intensive care unit (ICU). An insulin drip was initiated, and intravenous fluids were administered, but the patient's clinical condition continued to worsen. Simultaneously, a complete metabolic panel conducted upon admission revealed elevated liver enzymes, including aspartate aminotransferase (AST) of 667 U/L, alkaline phosphatase (ALP) of 523 U/L, and alanine transaminase (ALT) of 628 U/L. Due to the transaminitis, an ultrasound of the abdomen was ordered, which revealed a large irregular lesion measuring 11.3 x 13.5 x 12.0 cm in the inferior region of the right lobe of the liver, described as a mass in the right upper quadrant (RUQ). A computed tomography (CT) scan of the abdomen and pelvis with intravenous contrast (Figure 1) was subsequently conducted, revealing a multiloculated cystic mass that involved the majority of the right lobe of the liver.

Based on the findings, the radiologist recommended performing a biopsy to ascertain the etiology of the lesion, whether it was malignant or infectious. However, following this point, the patient's clinical condition rapidly deteriorated, leading to septic shock. He was intubated and required the use of multiple vasopressors. The biopsy of the lesion had to be postponed multiple times as the patient developed thrombocytopenia, with a platelet count of 13 k/µL. Disseminated intravascular coagulation was ruled out as fibrinogen levels were elevated, measuring 540 mg/dL. CT scans of the head and chest with contrast were performed, but no masses or metastases were detected. Despite attempts to optimize the platelet count for the biopsy over the following days, it remained unstable, resulting in multiple rescheduled procedures. A total of 16 units of platelets were administered. On the sixth day of admission, a decision was made to proceed with the drainage and biopsy of the lesion, considering the risk-to-benefit ratio favored intervention due to the suspected role of the lesion in driving sepsis. CT-guided drainage of the hepatic lesion confirmed it to be an abscess, and a pigtail catheter was inserted. Upon insertion, 400 ml of fluid with the consistency of anchovy paste was drained, and samples were sent to Pathology for analysis.

**Figure 2:**
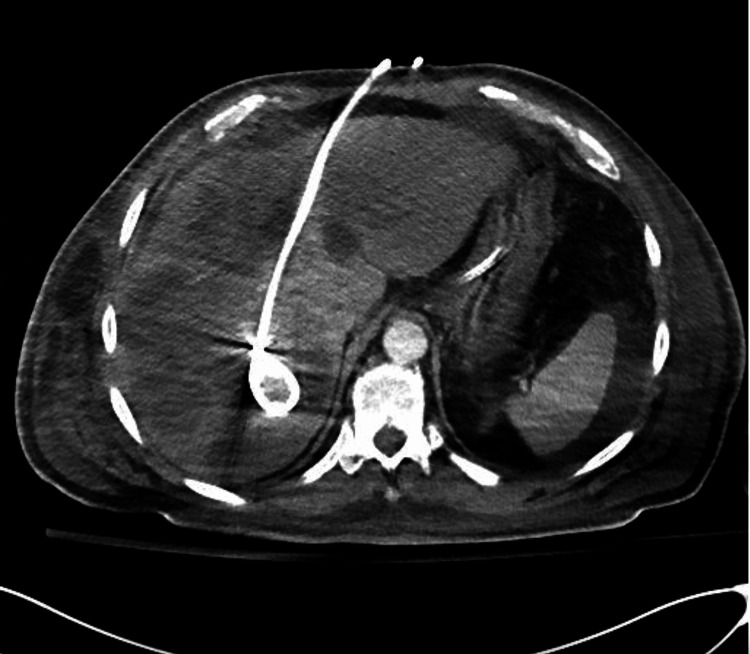
CT scan of the abdomen percutaneous drainage was performed with a catheter in place CT, computed tomography.

The patient was continued on meropenem and IV metronidazole. Blood cultures yielded positive results for *Escherichia coli*, while the abscess culture indicated the presence of both *E. coli* and *Stenotrophomonas maltophilia*. Following infectious disease recommendations, sulfamethoxazole and trimethoprim were added to the treatment regimen. However, despite these interventions, the patient's clinical condition remained critical without significant improvement. A repeat CT scan of the abdomen and pelvis revealed the persistent presence of multiseptated, multilocular confluent fluid containing abscess collections dispersed throughout the anterior and posterior segments of the right hepatic lobe. At this point, it became clear that the source of infection was not under control. On day 10, interventional radiology performed a second CT-guided drainage of a large liver abscess (Figure 2). A draining catheter connected to a collecting bag was placed inside the newly drained abscess, from which a significant amount of pus was aspirated. Despite these interventions, the patient's condition failed to improve. General surgery recommended hepatectomy on day 24 of admission, despite the patient being at a very high risk for intraoperative death. However, the patient continued to experience thrombocytopenia. Despite the risks involved, the patient's family opted for the procedure after it was explained that the extensive liver involvement made the patient at high risk. The patient was taken to the operating room for an open diagnostic laparoscopy with liver abscess drainage and an open right hepatectomy, with abthera vacuum placement. Unfortunately, during the procedure, the patient started to bleed heavily, leading to the termination of the intervention. Regrettably, the patient did not survive the procedure. 

## Discussion

Even though PLA is uncommon, there has been an increase in the number of cases each year. In our case, our patient had multiple risk factors for PLA, but his presenting symptoms were atypical. He did not show signs of abdominal pain, fever, nausea, or vomiting, which at first made it hard to distinguish abscess from malignancy. The patient's risk factors included incomplete screenings for colon cancer, diabetes mellitus, and prior use of a PPI at home before seeking hospital care.

Our patient presented with a multiloculated liver abscess (Figure 1), which is commonly observed in cases of hematogenous origin associated with enteric Gram-negative bacteria such as *K. pneumoniae* and, less frequently, *E. coli*. This bacterial etiology has been linked to the development of distant septic metastasis. Other common PLA-causing bacterial (Staphylococcus and Streptococcus species) and fungal (*Candida albicans*) causes are described as more likely to develop in patients with comorbidities such as diabetes mellitus or in immunocompromised hosts [6, 8]. In this patient's case, he had positive cultures for both *E. coli *and *S. maltophilia, *which are rare in causing multiloculated PLA. Due to this, the origin of this PLA was difficult to establish. Given the presence of multiple liver abscesses and the patient's various comorbidities (diabetes mellitus, PPI use, and lack of colorectal cancer screening), establishing the specific cause of this infection became even more complex.

The usual treatment approach for these cases should be initial coverage of the aforementioned organisms with antibiotics such as piperacillin-tazobactam, amoxicillin-clavulanic acid, or third-generation cephalosporins (cefotaxime, ceftriaxone) in combination with an aminoglycoside (gentamicin). If there is suspicion of *Entamoeba histolytica *infection, metronidazole should be added to the treatment regimen. Patients with abscesses larger than 3 cm should undergo surgical debridement or percutaneous drainage [9, 10]. It has been reported that surgical treatment (open drainage) for large multiloculated abscesses (>3 cm) has a 100% success rate, whereas percutaneous drainage with antibiotic therapy has a success rate of 33% in these cases [8, 10].

Appropriate treatment for this case was significantly delayed due to the patient’s thrombocytopenia. The patient’s condition failed to improve despite multiple percutaneous biliary drainages and appropriate antibiotic regimes. Furthermore, due to the large size and multitude of abscesses, we believe that instead of initially opting for antibiotic and percutaneous drainage, this patient would have benefitted from early surgical intervention to debride the majority of the affected area.

## Conclusions

PLA, although rare, can occur as complications in patients with common conditions such as diabetes. The increased incidence of PLA, along with their associated morbidity and mortality, raises questions about the optimal management of these cases. It prompts consideration of whether the benefits of emergent surgical interventions outweigh the risks in cases involving large PLA in acutely ill patients.
